# Effects of Prebiotic and Probiotic Supplementation on Lactase Deficiency and Lactose Intolerance: A Systematic Review of Controlled Trials

**DOI:** 10.3390/nu12051487

**Published:** 2020-05-20

**Authors:** Rosaura Leis, María-José de Castro, Carmela de Lamas, Rosaura Picáns, María L. Couce

**Affiliations:** 1 Department of Pediatrics, University Clinical Hospital of Santiago de Compostela, 15704 Santiago de Compostela, Spain; mj.decastrol@gmail.com (M.-J.d.C.); rosaurapicansleis@gmail.com (R.P.); maria.luz.couce.pico@sergas.es (M.L.C.); 2 IDIS-Health Research Institute of Santiago de Compostela, 15704 Santiago de Compostela, Spain; 3 CIBEROBN, Instituto Salud Carlos III, 28029 Madrid, Spain; 4 Facultad de Medicina, Departamento de Pediatría, Universidad de Santiago de Compostela, 15704 Santiago de Compostela, Spain; carmeladelamas@gmail.com; 5 CIBERER, Instituto Salud Carlos III, 28029 Madrid, Spain

**Keywords:** prebiotics, probiotics, lactose intolerance, hydrogen breath test, vomiting, diarrhea, flatulence, abdominal pain

## Abstract

Lactose intolerance (LI) is characterized by the presence of primarily gastrointestinal clinical signs resulting from colonic fermentation of lactose, the absorption of which is impaired due to a deficiency in the lactase enzyme. These clinical signs can be modified by several factors, including lactose dose, residual lactase expression, concurrent ingestion of other dietary components, gut-transit time, and enteric microbiome composition. In many of individuals with lactose malabsorption, clinical signs may be absent after consumption of normal amounts of milk or, in particular, dairy products (yogurt and cheese), which contain lactose partially digested by live bacteria. The intestinal microbiota can be modulated by biotic supplementation, which may alleviate the signs and symptoms of LI. This systematic review summarizes the available evidence on the influence of prebiotics and probiotics on lactase deficiency and LI. The literature search was conducted using the MEDLINE (via PUBMED) and SCOPUS databases following Preferred Reporting Items for Systematic Reviews and Meta-Analyses (PRISMA) guidelines, and included randomized controlled trials. For each study selected, the risk of bias was assessed following the Cochrane Collaboration methodology. Our findings showed varying degrees of efficacy but an overall positive relationship between probiotics and LI in relation to specific strains and concentrations. Limitations regarding the wide heterogeneity between the studies included in this review should be taken into account. Only one study examined the benefits of prebiotic supplementation and LI. So further clinical trials are needed in order to gather more evidence.

## 1. Introduction

Lactose intolerance (LI) is one of the most common forms of food intolerance and occurs when lactase activity is reduced in the brush border of the small bowel mucosa [1,2]. It is characterized by the presence of gastrointestinal symptoms including vomiting, diarrhea, flatulence, and abdominal pain, which are caused by colonic fermentation of unabsorbed lactose [3,4]. The severity of LI, and of the aforementioned symptoms, can vary considerably between individuals.

Lactase deficiency can be primary, secondary, or congenital. The most frequent form is primary lactase deficiency, a consequence of lactase non-persistence characterized by a progressive decline in lactase activity [5]. The prevalence of adult-type lactase deficiency varies among different ethnic groups and geographic locations (following a north–south gradient), ranging from 5%–15% in Northern Central Europe and North American countries to 40% in Mediterranean countries and 65%–90% in African, Asian, and South American countries [6,7,8]. In Europe, it is related to the presence of two single nucleotide polymorphisms (SNPs), C/T-13910 and G/A-22018, which mediate lactase downregulation after infancy [9]. Secondary lactase deficiency is caused by pathologies (e.g., celiac disease, Crohn’s disease, or infection) and procedures (e.g., surgery) that affect the small intestine and induce a loss of enzyme activity [10,11,12]. Congenital lactase deficiency is characterized by the total absence of lactase activity [13]. This form is extremely rare and manifests at birth, soon after the introduction of milk. Lactase levels are minimal or absent in affected infants, which have an otherwise normal intestinal mucosa. Gastrointestinal mucosal biopsy is the gold standard for the diagnosis of lactase deficiency, although the hydrogen breath test (HBT) is also commonly used [14,15]. Bacterial fermentation of undigested and unabsorbed lactose leads to an increase in exhaled hydrogen. A deficit in the lactase enzyme leads to lactose malabsorption, since the disaccharide cannot be absorbed and is instead fermented by gut microbiota, leading to the development of clinical signs characteristic of LI. Management of LI typically consists of reducing, or even avoiding, the consumption of dairy products [16,17]. However, because dairy products constitute a high-quality source of calcium, potassium, protein, and vitamin B and D, avoidance of these foods can increase the risk of morbidity, including bone fracture, osteoporosis, and nutrient deficiencies [18,19,20]. The most preferred and reliable treatment option involves the consumption of lactose-free dairy products. Furthermore, lactase enzyme supplementation in tablet form [21] can be employed in a timely manner when ingesting products with lactose. However, the effects of exogenous lactase administration in reducing LI symptoms vary considerably [22].

Clinical symptomatology in LI is modified by several factors, including the load of the lactose substrate, lactase activity, the speed of intestinal transit, the rate of gastric emptying, and colonic compensation [1,2,23]. Probiotics and prebiotics have attracted considerable interest in recent years as potential symptomatic treatments for lactase insufficiency, owing to their ability to modulate the gastrointestinal flora, promoting lactase digestion and increasing colonic compensation [24,25]. Indeed, consumption of yoghurt containing live bacterial cultures has been shown to ameliorate maldigestion and symptoms in lactase-deficient individuals. Moreover, consumption of fresh rather than pasteurized yoghurt is associated with improved lactose absorption [26]. Nonetheless, it should be borne in mind that unabsorbed lactose may constitute a good prebiotic, helping to maintain a healthier intestinal flora [27]. It should be noted that probiotics and prebiotics supplementation would not be a substitute for the lactose-free products.

Despite the aforementioned findings, data on the relationship between prebiotic and/or probiotic supplementation and to clinical outcomes in LI individuals remain inconclusive. This systematic review presents an updated evaluation of the available evidence from clinical trials (CT) assessing the impact of this type of intervention on the clinical signs of LI and on HBT results in lactase-deficient individuals.

## 2. Materials and Methods 

This review was carried out following Preferred Reporting Items for Systematic Reviews and Meta-Analyses (PRISMA) guidelines [28], and was registered in the International Prospective Register of Systematic Reviews (PROSPERO). The review question, “Do biotics influence lactose intolerance?” was formulated according to Population, Intervention, Comparison, Outcome, Settings (PICOS) [29] criteria (Table 1).

### 2.1. Literature Search

The articles included in this review were selected from SCOPUS and PUBMED databases. Moreover, a manual search of the reference list of included ones was done in order to ensure that all eligible studies were selected. (“*Lactobacillus*” [Mesh] OR “*Bifidobacterium*” [Mesh] OR “*Saccharomyces boulardii*” [Mesh] OR “*Streptococcus thermophilus*” [Mesh] OR “Prebiotics” [Mesh] OR “Probiotics” [Mesh]) AND “Lactose Intolerance” [Mesh] was the PUBMED search formula used. The Scopus database was searched using the following formula: “Lactose Intolerance” AND (“*Lactobacillus”* OR “*Bifidobacterium”* OR “*Saccharomyces boulardii*” OR “*Streptococcus thermophilus*” OR Prebiotics OR Probiotics). 

### 2.2. Inclusion and Exclusion Criteria

Articles considered for inclusion were any controlled trial, randomized or not, published in English or Spanish between 1 January 1900 and 31 December 2019. All studies of LI patients of any age and ethnicity who underwent an intervention with prebiotics and/or probiotics were considered. The following exclusion criteria were applied: Patients with chronic diseases and/or studies that combine the consumption of biotics with other non-biotic elements, not controlled in some way by an independent arm, that could interfere with the results. 

### 2.3. Intervention Types

Studies considered for inclusion were those involving interventions with prebiotics and/or probiotics in populations with altered lactose absorption. Any study that met these characteristics, regardless of duration, intensity, or type of biotic used, was considered for inclusion. 

### 2.4. Primary Outcome Measures

The primary outcome measures of lactose metabolism were the concentration of exhaled hydrogen after lactose intake and the percentage of patients with normalized HBT results. For the assessment of gastrointestinal symptomatology, we considered all studies that provided data on symptom improvement, either using standardized scales for symptom measurements or by measuring symptom disappearance. 

### 2.5. Study Selection

Two authors (M.-J.d.C. and C.d.L.) independently selected the nine articles [30,31,32,33,34,35,36,37,38], ultimately, included in the review from a total of 633 studies obtained by database searches. In cases in which no consensus was reached, R.L., R.P., and M.L.C. acted as arbitrators. 

### 2.6. Data Extraction

The two authors independently extracted the following data from the selected articles: Publication year; number of participants by sex, age, intervention characteristics, and treatment duration; trial type; outcome measures; results; and conclusions. Any discrepancies were arbitrated by the remaining authors. 

### 2.7. Assessment of Risk of Bias

Following the methodology of The Cochrane Collaboration, London, UK [39], two evaluators independently assessed the risk of bias in each study. For each study, each of the following risks of bias were assessed: Selection bias (random sequence generation, allocation concealment); performance bias (blinding of participants and personnel); detection bias (blinding of outcome assessment); attrition bias (incomplete outcome data); reporting bias (selective reporting); and any other forms of bias. For each study, the risk of each type of bias was classified as low, high or, in cases in which insufficient data were reported, unclear. In cases of a lack of consensus, R.L. and M.L.C. acted as arbitrators. 

## 3. Results

**Figure 1:**
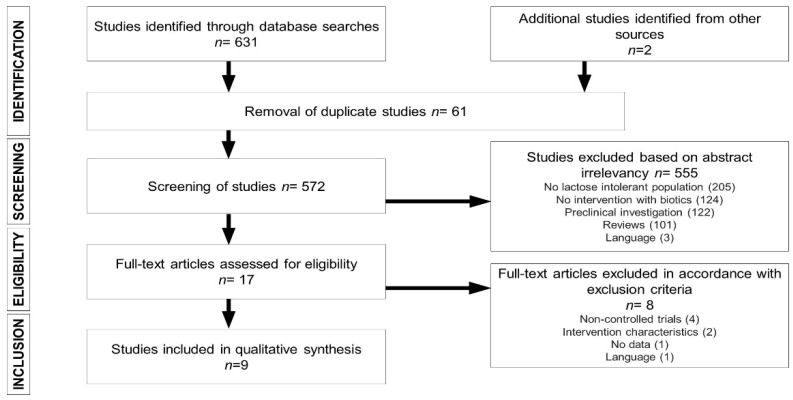
Flow chart depicting literature search process.

Figure 1 summarizes the process by which articles were selected for this systematic review. The SCOPUS search generated 528 articles, while de PUBMED search allowed us to obtain 45 more studies. Two more articles, from the manual review of the bibliography of the participating articles, were included. Of the 633 articles identified in database searches, 61 duplicate articles were excluded, and 555 were excluded due to a lack of relevance of the abstract (205 did not include a LI population, 124 lacked a biotic intervention, 122 were preclinical studies, 101 were systematic or narrative reviews, and three were published in languages other than English or Spanish). Of the 17 full-text articles reviewed, four were excluded due to the lack of a control group; two due to unsuitable intervention characteristics; one due to the absence of data on gastrointestinal symptoms or lactose metabolism; and one due to the publication language. Ultimately, nine [30,31,32,33,34,35,36,37,38] articles were selected for inclusion in this systematic review.

### 3.1. Study Characteristics

Table 2 and Table 3 summarize the main characteristics of the nine [30,31,32,33,34,35,36,37,38] selected randomized clinical trials (RCTs), which are ordered according to the age of the study population. Five crossover trials were included [30,31,35,36,38]. All studies [30,31,32,33,34,35,36,37,38] were published after 1983, and five [31,32,33,34,35] in the last 10 years. In total, the nine studies included 304 LI patients, of whom 111 participated in crossover clinical trials. The age of the study populations ranged from 5 to 75 years, and only one study [30] involved a pediatric population. One of the included studies [34] involved an intervention with prebiotics (15 g RP-G28 (95% GOS)/day in capsules). Of the eight studies [30,31,32,33,35,36,37,38] involving probiotic interventions, three [31,33,37] consisted of an intervention with a single strain and five [30,32,35,36,38] with two or three strains. All of the probiotic interventions [30,31,32,33,35,36,37,38] involved some species of *Lactobacillus*: *L. acidophilus* (5) [30,31,36,37,38]; *L. bulgaricus* (2) [36,38]*; L. plantarum* (1) [32]; *L. reuteri* (1) [33]; or *L. rhamnosus* (1) [35]. Other strains studied included *Bifidobacterium animalis* [32], *Bifidobacterium longum* [35], and *Streptococcus thermophilus* [30,38]. The dose of probiotic used ranged from 10^7^ [38] CFU (colony-forming units) to 10^10^ CFU [30,32]. Three [30,36,38] of the studies included in the review, all of which were crossover studies, were based on a punctual intervention, while in the remaining six [31,32,33,34,35,37] the duration of the intervention ranged from six days to six weeks (mean, 24.83 ± 12.86 days). 

### 3.2. Prebiotics, Probiotics, and LI Symptoms

Seven [30,31,32,33,34,35,36] of the articles included in this systematic review assessed the effects of prebiotics or probiotics on symptoms of LI in 170 subjects with lactose malabsorption after ingestion of 2–50 g/kg of lactose. Four of the studies were crossover RCTs [30,31,35,36]. The study involving prebiotic supplementation [34] evaluated rate of disappearance of abdominal pain. The remaining six studies [30,31,32,33,35,36] evaluated gastrointestinal symptoms using different standardized scales (of 0–4 or 0–100) to rate symptom intensity. Only one [32] of these seven studies [30,31,32,33,34,35,36] reported no significant effect of the intervention. The study in question conducted the longest intervention (six weeks), using a high daily dose of probiotics (10^10^ CFU *L. plantarum* + 10^10^ CFU *B. animalis*). However, in this same study an evaluation performed two weeks after completion of the intervention revealed that probiotic supplementation was associated with a significant decrease in diarrhea and flatulence. 

### 3.3. Prebiotics, Probiotics, and Lactose Digestion

Of the nine [30,31,32,33,34,35,36,37,38] studies included in this review, only five [33,34,36,37,38], accounting for 179 individuals with lactose malabsorption, included data on lactose maldigestion after ingestion of 20–50 g of lactose. In order to reduce external factors in HBT and symptom results, the included studies followed a standardized method, consisting of dietary restrictions for the previous days, low intake of sugar, carbohydrates, and fiber, and fasting for 8–10 h before the lactose challenge. In these five studies, lactose metabolism was evaluated by HBT. Two of the five studies [36,38] were crossover RCTs consisting of a punctual probiotic intervention before the ingestion of 25 g of lactose. The only study [34] that reported no significant effect of the intervention was the one in which participants underwent a prebiotic intervention. Studies comparing different doses of probiotics [36,37,38] observed significant differences between the effects of high doses and the effects of lower doses. 

### 3.4. Risk-of-Bias Assessment

Risk-of-bias assessment revealed that none of the studies included [30,31,32,33,34,35,36,37,38] had a high risk of selection bias, and that the risk of random sequence generation was low in all cases. The risk of reporting bias was uncertain for all nine studies [30,31,32,33,34,35,36,37,38]. A high risk of attrition bias was observed for only one study [30], and a high risk of performance bias for two (22%) [30,33]. A high risk of detection bias was observed for two (22%) [30,38] studies. The risk of other biases was high in the five crossover studies [30,31,35,36,38], due to the high risk of carry-over, and in one multicenter study [34], which lacked standardized protocols. 

The study with the highest risk of biased results was that of Montes et al. [30], for which the level of risk was deemed low for only one of the seven forms of bias assessed. Our analyses revealed that there was no high risk of any of the forms of bias assessed in two studies [32,37]. In the six [31,33,34,35,36,38] remaining articles, the risk of bias was deemed high for only one [31,33,34,35,36] or two [38] of the forms of bias assessed. Further information on the risk-of-bias assessment can be found in the Supplementary Materials. 

## 4. Discussion

This systematic review of RCTs assesses the effects of probiotic or prebiotic supplementation on HBT results and on the clinical signs of LI. Probiotic supplementation improved both outcomes in patients with LI. Prebiotic supplementation, which was assessed in only one study [34], had a beneficial effect on clinical signs of LI but not on HBT results.

Evaluation of the RCTs included in this review revealed that probiotic supplementation in individuals with LI significantly reduced abdominal cramping, diarrhea, vomiting, bloating, and/or flatulence. This effect, together with the reduction in exhaled H2, may be explained by several mechanisms. First, upon reaching the digestive system probiotics act as a source of lactase in the intestinal tract [40], increasing the overall hydrolytic capacity and colonic fermentation [41]. Second, probiotics exert antagonistic effects on heterofermentative bacteria (which produce gas), enhancing colonic compensation [42] by secreting antibiotic-like substances [43], adhering competitively to the mucosa, and modulating the permeability of the intestinal barrier [44,45]. Other mechanisms, like decreasing lactose load [46] and delaying gastric emptying and orocecal transit time, which are dependent on the accompanying matrix, should be irrelevant [47]. 

Common criteria used to select the genus, species, and strains of probiotic microorganisms include tolerance to the intestinal environment, capacity to adhere to the intestinal mucosa, and competitive exclusion of pathogens [48]. In the nine studies evaluated here, most of the species of probiotics administered (including *L. acidophilus, L. reuteri, L. rhamnosus, and L. bulgaricus*, *S. thermophilus,* and *B. longum*) were effective in attenuating clinical signs. Only one study [32], in which *L. plantarum* and *B. animalis* were administered, observed no significant effect of the probiotic on clinical signs. However, beneficial effects of both microorganisms have been demonstrated both in vitro and in vivo. Specifically, *B. animalis* is one of the most common bacteria found in gut microbiota and one of the best studied probiotic bifidobacteria; this bacterium has strong mucus adherence properties, inhibits pathogens, and improves barrier function [49], as well as enhancing lactose digestion and increasing transit time in patients with LI [50]. The lack of effect of this probiotic may be explained by the fact that different types and different concentrations of probiotics may exert different effects, and only specific combinations of probiotics may be effective in alleviating symptoms, in line with the global guidelines of the World Gastroenterology Organisation [https://www.worldgastroenterology.org/UserFiles/file/guidelines/probiotics-and-prebiotics-english-2017.pdf].

Prebiotics are functional foods that stimulate the growth of beneficial native gut bacteria and increase colon permeability [51], potentially mitigating the symptoms of LI. Specifically, galacto-oligosaccharides (GOS) have been shown to increase the abundance of lactose-fermenting *Bifidobacterium, Faecalibacterium, Lactobacillus,* and *Roseburia* species in the gut [52]. It should be noted that mechanisms of GOS utilization by intestinal bacteria are not fully understood, and efficacy and response vary between strains [53].

Lactase deficiency and lactose malabsorption in humans is usually assessed using the HBT [1], which measures the concentration of exhaled H_2_ after ingestion of lactose. The increase in exhaled H_2_ results from the release of gases by heterofermentative bacteria that digest lactose [53]. However, the correlation between lactose malabsorption (or the load of undigested lactose) and LI (presence of symptoms) is not always clear: The HBT is thought to produce 5%–15% false negatives, mainly due to non-hydrogen production and methane production [54,55,56]. 

While one study included in this systematic review found no significant decrease in exhaled H_2_ concentration following a probiotic intervention, the authors did observe a significant decrease in abdominal pain [34]. This may be explained by the fact that gases are partially responsible for the symptoms of LI. In a study of 30 self-described “severely lactose intolerant individuals”, Suarez et al. [57] found that HBT values were normal in 30% of the participants. This percentage exceeds the estimated rate of false negatives due to methane production and suggests a role of other pathophysiological mechanisms in LI (e.g., an osmotic effect caused by the presence of lactose molecules in the gastrointestinal tract) [58]. Furthermore, patient-related factors not directly related to lactose digestion are also implicated in LI. These include anxiety, high levels of psychosocial stress, and functional gastrointestinal disorders such as irritable bowel syndrome [59].

It should be noted that dose is a critical parameter when administering probiotics, as the changes that occur in the composition of the microflora depend on the minimum number of microorganisms required for colonization [60]. The importance of dose selection has also been emphasized by the joint working group of the FAO/WHO (2002), which recommended defining probiotics as “live microorganisms which when administered in adequate amounts confer a health benefit on the host”. Most of the studies included in this review tested only a single dose of probiotics, ranging from 10^8^ –10^11^ CFU/day. Only one study (Lin et al. 1998) compared two different doses (4 × 10^8^ and 4 × 10^9^ CFU/day) for two different *Lactobacillus* species (*L. acidophilus* and *L. bulgaricus*), and reported a dose-dependent effect on clinical signs of LI. In measuring the effects on HBT, one study (Lin et al. 1991) compared different doses (10^7^–10^8^ CFU/day) for three different *L. acidophilus* strains (NCFM, LA1, and LA2) and the combination of *S. thermophilus* and *L. bulgaricus*, and reported better results with higher doses.

Another factor that should be taken into account is the duration of the intervention and its impact after discontinuation. This issue was addressed in a study of individuals with IL who underwent a four-week probiotic intervention consisting of a combination of *Lactobacillus casei Shirota* and *Bifidobacterium breve Yakult* [61]. The intervention improved symptoms and decreased the concentration of exhaled hydrogen, and the effects persisted for at least three months after discontinuation. However, data from patients with other gastrointestinal diseases, such as irritable bowel syndrome, suggest that the effects of probiotics may wane several weeks after discontinuation [62]. Because LI is a chronic condition and changes in gut microbiota may not persist, long-term efficacy trials, particularly those that examine effects after cessation of treatment, are needed.

The limitations of this review are related to the high heterogeneity of the selected studies, including the age of the participants (one study was conducted in children, one study did not mention the age of the participants and the remaining ones were conducted in adults). Intestinal microbiota varies with age [63], which implies that results may not be comparable between children and adults. Another limitation is the wide variation of the duration of the intervention, ranging from a timely intervention to six weeks probiotics administration. Given that duration of the intervention is a key factor to see changes in gut flora following probiotics and prebiotics consumption, longer interventions are desirable in order to ensure that a potential effect can be observed.

## 5. Conclusions

The findings of this systematic review support the beneficial effects of probiotic supplementation on HBT results and on LI symptoms, as evidenced by decreases in the concentration of exhaled hydrogen and reductions in abdominal cramping, diarrhea, vomiting, bloating, and/or flatulence. Further long-term trials should be conducted in order to determine the persistence of the beneficial effects of probiotic administration and whether symptoms worsen after discontinuing supplementation. Based on our findings, evidence supporting the beneficial effects of prebiotic supplementation remains inconclusive. 

## Figures and Tables

**Table 1:** Population, Intervention, Comparison, Outcome, Settings (PICOS) criteria [29] for the inclusion of studies of the effects of prebiotics and probiotics on lactose intolerance.

Parameter ^1^	Inclusion Criteria
Population	Lactose-intolerant subjects
Intervention	Controlled intake of biotics
Comparison	Non-exposed control group
Outcomes	Symptoms of lactose intolerance and signs of lactose malabsorption
Settings	Controlled trials

^1^ PICOS criteria [28].

**Table 2:** Effects of prebiotics and probiotics on symptoms of lactose intolerance in 170 individuals with lactose malabsorption in controlled trials.

Reference	*n*	Age, y ^1^	Intervention	Trial Type (Intervention Duration)	Outcome Measure	Results ^2^	Conclusions
Montes et al. (1995) [30]	20 (11F)	5–16	IG1: 10^10^ CFU *L. acidophilus* IG2: 10^8^ CFU *L. acidophilus* + 10^10^ CFU *S. thermophilus* (250 mL milk)	Crossover RCT (-)	Mean 8-h symptom score for abdominal pain, bloating, borborygmi and flatus (0 = absent, 4 = severe symptoms) after ingestion of 2 g/kg of lactose	Symptom score: IG1 0.9 ± 0.43; IG2 1.62 ± 0.71; CG 4.6 ± 0.73	Significantly lower symptom score
Pakdaman et al. (2016) [31]	38	18–75	10^9^ CFU DDS-1 strain of *L. acidophilus*/day (capsules)	Crossover RCT (4 weeks)	Mean 6-h symptom scores (0 = no symptoms, 10 = most severe symptoms) after ingestion of 25 g of lactose	Abdominal cramping: IG 1.94 ± 2.341; CG 2.39 ± 2.188 Bowel sounds: IG 2.76 ± 2.536; CG 2.86 ± 2.497 Diarrhea: IG 1.34 ± 2.462; CG 1.69 ± 2.558 Flatulence: IG 3.16 ± 2.873; CG 3.21 ± 2.699 Vomiting: IG 0.08 ± 0.379; CG 0.36 ± 0.936 Overall symptoms: IG 9.28 ± 9.202; CG 10.51 ± 9.327	Significantly less abdominal cramping, diarrhea, vomiting and lower overall symptom score
Roškar et al. (2017) [32]	44 (36F) IG 22	IG 28 (19–54) CG 31 (18–55)	10^10^ CFU *L. plantarum* + 10^10^ CFU *B. animalis*/day (capsules)	RCT (6 weeks)	Mean LI symptom assessment score (0 = absent, 10 = worst)	Abdominal pain: IG 2.4 (1.3–3.4); CG 2.3 (0.9–3.7) Diarrhea: IG 0.3 (−0.1;0.8); CG 0.6 (−0.3;1.5) Flatulence: IG 4.2 (2.9–5.5); CG 4.2 (2.8–5.5) Rumble: IG 3.9 (2.8–5.1); CG 3.6 (2.1–5.1) Vomiting: IG 0.2 (−0.2;0.7); CG 0.2 (−0.1;0.4) Total (Ʃ): IG 11.1 (7.9–14.3); CG: 10.8 (6.4–5.3)	No significant differences
Ojetti et al. (2010) [33]	40 (33F) IG 20	IG 33 ± 11 CG 32 ± 12	8 × 10^8^ CFU *L. reuteri*/day (capsules)	RCT (10 days)	Mean 8-h symptom scores values (0 = absent, 10 = severe symptoms) after ingestion of 25 g of lactose	Abdominal pain: IG 6.9 ± 1.07; CG 7.1 ± 0.72 Bloating: IG 9.95±0.88; CG 7.1 ± 0.72 Diarrhea: IG 2.95±2.07; CG 5.9 ± 0.85 Flatulence: IG 3.95 ± 1.35; CG: 5.15 ± 0.93	Significant improvement in abdominal pain, bloating, diarrhea, and flatulence
Savaiano et al. (2013) [34]	85 (49F) IG 57	41	15 g RP-G28 (95% GOS)/day (capsules)	RCT (35 days)	Rate of disappearance of abdominal pain (%)	Abdominal pain: IG 72%; CG 28%	Significantly higher rate of disappearance of abdominal pain
Vitellio et al. (2019) [35]	23 (19F)	48 ± 3.1	4 × 10^9^ CFU *B. longum* BB536 + 10^9^ CFU *L. rhamnosus/*day (packets)	Crossover RCT (4 weeks)	Mean VAS perceived symptom score (0 = absent, 100 = worst) [abdominal pain and bloating] and mean BSFS (1 = constipation, 7 = diarrhea)	Abdominal pain: IG 39 ± 6; CG 53 ± 7 Bloating: IG 60 ± 5; CG 77 ± 4 Bowel movements: IG 3 ± 0; CG 3 ± 0	Significantly less bloating
Lin et al. (1998) [36]	20	-	IG1: 4 × 10^8^ CFU *L. acidophilus*/day IG2: 4 × 10^9^ CFU *L. acidophilus*/day IG3: 4 × 10^8^ CFU *L. bulgaricus*/day IG4: 4 × 10^9^ CFU *L. bulgaricus*/day (400 mL milk)	Crossover RCT (-)	Mean 8-h symptom score for stomach pain, gas, and diarrhea (0 = absent, 5 = severe) after ingestion of 25 g of lactose	Symptom score: IG1 9.8; IG2 6.5; IG3 3.9; IG4 2.8; CG 12.5	Significantly lower symptom score in IG2, IG3, and IG4

*B*., *Bifidobacterium*; BSFS, Bristol stool form scale; CFU, colony-forming unit; CG, control group; F, female; GOS, galacto-oligosaccharide; h, hours; IG, intervention group; *L*., *Lactobacillus*; RCT, randomized controlled trial; *S*., S*treptococcus*; and VAS, visual analogue scale. ^1^ Values represent the range, the mean (95% CI), or the mean ± SD in years, as reported in the corresponding article. ^2^ Values represent the mean, mean ± SD, or mean (range) as reported in the corresponding article.

**Table 3:** Effects of prebiotics and probiotics on lactose digestion in 179 individuals with lactose malabsorption in controlled trials.

Reference	*n*	Age, y ^1^	Intervention	Trial Type (Intervention Duration)	Outcome Measure	Results ^2^	Conclusions
Kim et al. (1983) [37]	24 IG 6 × 3	20–31	IG1: 1.25 × 10^7^ CFU *L. acidophilus*/kg/day IG2: 1.25 × 10^8^ CFU *L. acidophilus*/kg/day IG3: 1.25 × 10^9^ CFU *L. acidophilus*/kg/day (milk 10 mL/kg/day)	RCT (6 days)	Change in mean breath H concentration (ppm) 3 h after ingestion of 5 mL/kg milk	Change in mean breath H concentration: IG1-15.2; IG2-1.1; IG3-19.2; CG-0.3	Significant change in mean breath H concentration in IG1 and IG3.
Lin et al. (1991) [38]	10 (4F)	24–40	IG1: 10^7^ CFU *L. acidophilus* NCFM/day IG2: 10^8^ CFU *L. acidophilus* NCFM/day IG3: 10^7^ CFU *L. acidophilus* LA1/day IG4: 10^8^ CFU *L. acidophilus* LA1/day IG5: 10^7^ CFU *L. acidophilus* LA2/day IG6: 10^8^ CFU *L. acidophilus* LA2/day IG7: 10^7^ CFU *S. thermophilus*/*L. bulgaricus*/day IG8: 10^8^ CFU *S. thermophilus/L. bulgaricus*/day (400 mL milk)	Crossover RCT (-)	Mean individual breath H concentration 8 h after ingestion of 25 g lactose	Breath H concentration: IG1 36.33; IG2 35.08; IG3 27.64; IG4 22.43; IG5 31.03; IG6 25.32; IG7 24.1; IG8 9.81; CG 30.78	Significantly lower breath H concentration in IG4 and IG8
Ojetti et al. (2010) [33]	40 (33F) IG 20	IG 33 ± 11 CG 32 ± 12	8 × 10^8^ CFU *L. reuteri*/day (capsules)	RCT (10 days)	HBT normalization rate (%) Mean peak H_2_ excretion (ppm)	HBT normalization rate: IG 35%; CG 0% Peak H_2_: IG 23.1 ± 7.85; CG 31.7 ± 8.3	Significantly higher HBT normalization rate and reduced mean peak H_2_ excretion
Savaiano et al. (2013) [34]	85 (49F) IG 57	41	15 g RP-G28 (95% GOS)/day (capsules)	RCT (35 days)	Mean change in HBT values 2 h after ingestion of 25 g lactose	HBT change: IG-10.12; CG 13.95	No significant differences
Lin et al. (1998) [36]	20	-	IG1: 4 × 10^8^ CFU *L. acidophilus*/day IG2: 4 × 10^9^ CFU *L. acidophilus*/day IG3: 4 × 10^8^ CFU *L. bulgaricus*/day IG4: 4 × 10^9^ CFU *L. bulgaricus*/day (400 mL milk)	Crossover RCT (-)	Mean hourly breath H concentration 8 h after ingestion of 25 g lactose	Breath H: IG1 262; IG2 231; IG3 188; IG4 135; CG 280	Significantly lower breath H concentration in IG3 and IG4 (*L. bulgaricus*)

CFU, colony-forming unit; CG, control group; F, female; GOS, galacto-oligosaccharide; H, hydrogen; HBT, hydrogen breath test; h, hours; IG, intervention group; *L.*, *Lactobacillus*; p, parts per million; RCT, randomized controlled trial; and *S*., *Streptococcus*. ^1^ Values represent the mean or the mean ± SD, as reported in the corresponding article. ^2^ Values represent the mean, mean changes, or normalization rate (%), as reported in the corresponding article.

## Supplementary Materials

The following material is available online at https://www.mdpi.com/2072-6643/12/5/1487/s1. Figure S1, Risk-of-bias summary: Summary of the authors’ rating on each risk-of-bias item for each study. Figure S2, Risk-of-bias graph: Review of the authors’ judgments on each risk-of-bias item, expressed as percentages across studies.
