# Supplementary material for: Effects of Prebiotic and Probiotic Supplementation on Lactase Deficiency and Lactose Intolerance: A Systematic Review of Controlled Trials
Source: Nutrients. 2020 May 20;12(5):1487. doi: 10.3390/nu12051487 (PMC7284493; doi:10.3390/nu12051487)
Supplement: Supplementary file 1 [file nutrients-12-01487-s001.pdf]

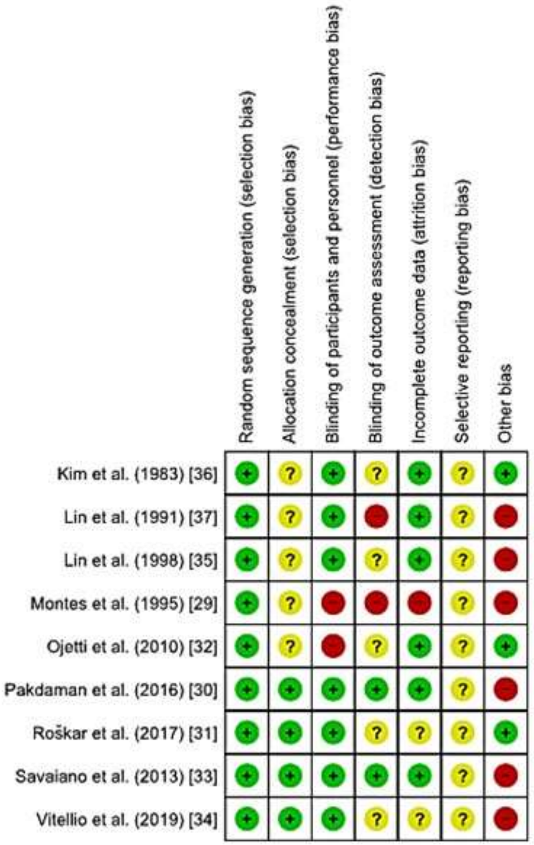

Supplemental Figure 1. Risk of bias summary: review of authors' judgements on each risk of bias item for each included study

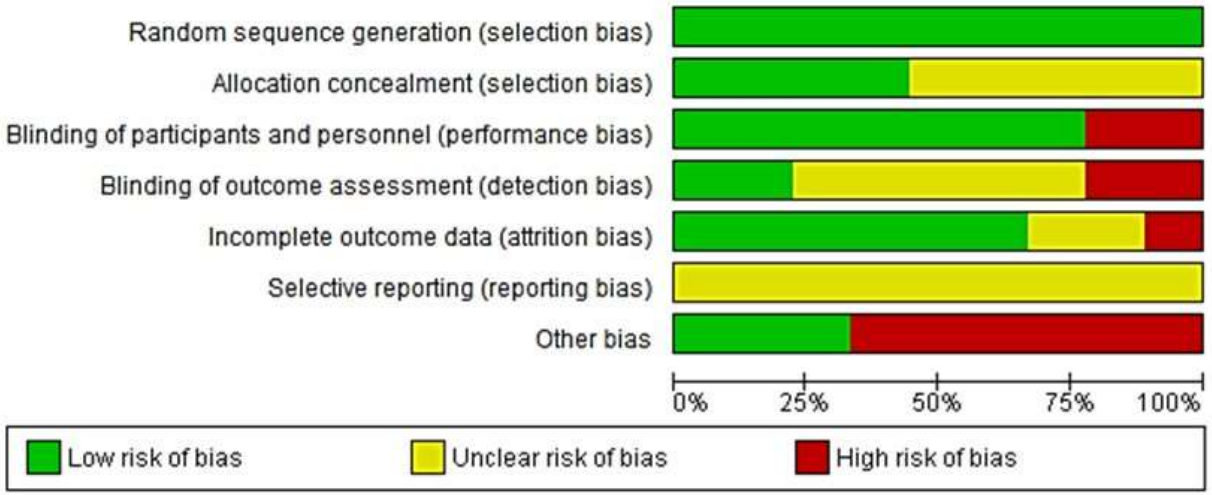

Supplemental Figure 2. Risk of bias graph: review of authors' judgements on each risk of bias item presented as percentages across the studies
